# The reality of the dead in Brazil: perspectives on identification in forensic anthropology

**DOI:** 10.1080/20961790.2022.2125414

**Published:** 2023-02-12

**Authors:** Eugénia Cunha, Bridget Algee-Hewitt, Melina Calmon

**Affiliations:** a National Institute of Legal Medicine and Forensic Sciences, Coimbra, Portugal; b Centre for Functional Ecology, Department of Life Sciences, University of Coimbra, Coimbra, Portugal; c CCSRE Center for Comparative Studies in Race and Ethnicity (CCSRE), Stanford University, Stanford, CA, USA; d Grupo de Pesquisa em Antropologia Forense e Identificação de Pessoas – ANP/CNPq, Lago Sul, Brazil

What is the current state of Forensic Anthropology in Brazil and where do we stand when identification is concerned? Coming to terms with the *reality of identifying the dead in Brazil* is the challenge we proposed to several Brazilian forensic anthropology experts and it is the focus of this special issue. We asked the contributor to drill-down to the fundamental questions in order to define the baseline upon which we can build our understanding and establish the future pathways for the field. What are the most typical cases? Is there a more frequent case type? Does the way the issues are being solved reflect advancements in the discipline in the country? Are standards procedures being followed? What are the main obstacles faced by the experts in this field?

This special issue compiles papers from experts from five different states: Minas Gerais (3 papers); ­Rio de Janeiro; Distrito Federal (3 papers); Santa Catarina; and Paraíba, shedding light on the forensic anthropology perspective of practice in the identification of the deceased in the country.

The experts invited were asked to tell us how their institutions adapted to the new challenges of this century and what they are doing in terms of groundbreaking research.

This special issue was born on an idea based on the knowledge of one of the authors (EC) regarding the forensic reality of Brazil. After more than 20 years of on-site work, she felt the need to share what was being done in the country. Because of that, several Brazilian experts were invited to participate and to present a typical case related to the reality of forensic anthropology in Brazil, whose major issues/difficulties will spark discussion. To this end, it was presented to inspire a debate on the larger set of challenges doing forensic/human rights work in Brazil and the reasons that make pursuing this work so important. Invited authors were forensic anthropology practitioners in Brazil representing the main cities in which practice is being pursued. They were asked to present a paradigmatic case that highlights the difficulties that they are faced with in general when performing casework. Likewise, they were also asked to explain, more specifically, the problems that this particular case has posed in terms of its assessment and identification, offering suggestions for why it is/was a complicated, hard case to resolve; and to discuss the strategies (realistic or wishful) that they believe may be helpful to overcome these challenges now and in the near future. Above all, we hoped to show the Reality of Forensic Anthropology in Brazil, specifically in the context of identification of the dead. Furthermore, we aimed to stimulate research and scientific publications.

Two main achievements resulted from this launched challenge. First, a symposium—unfortunately held online due to COVID-19 restrictions in 2020—with a large number of participants and where the presentations achieved a good quality, illustrating the context and cases of six different Brazilian states, sponsored by Centre for Latin American Studies, from Stanford University, where the senior guest editor (EC) held a fellowship. The success of the symposium led to the idea of this publication: there was an obvious need to disseminate what was being done in Brazil regarding forensic anthropology and create a discussion around these particularities. This second challenge was accepted by the large majority of the symposium presenters. It took more time than previewed, in part because the practice of publishing peer review articles in Brazil in this field is not well established. *Forensic Sciences Research* was the optimal recipient with a strong peer review system, which led to the rejection of four of the planned articles. Yet, we believe that the ones now published fulfill the requirements and are successfully representative.

The remarkable work done by the team of Belo Horizonte with the mass disaster due to the collapse of a mine tailing dam in Brumadinho, stands out for their main achievements [1]. The fact that most of the victims, more than two hundred, were identified in 1 year shows the quality of the forensic work. From the Southern State of Brazil, Santa Catarina, comes a very good article by Paulo Miamoto and collaborators [2]. It discusses personal identification and missing person initiatives in that state from 2019 to 2021, highlighting the recent advances and current challenges, namely the creation, in 2020, of a Forensic Anthropology Sector. It is worthwhile to detach their perspective since it does show the reality of Brazil. Subscribing these authors, there is a Brazilian paradox: a country that urges forensic work has legislation that severely restricts the professionals performing postmortem examinations. So far, forensic anthropologists are not previewed in the current federal or state legislation.

Silva [3], from Brasília, debates forensic data management and database systems in forensic investigations for cases of missing and unidentified persons in Brazil. This article seeks to approach some of the most critical components of an effective forensic data management system. Mainly, it aims to bring attention to the urgent need for an effective and integrated approach in Brazil.

Also from Brasília, Damasceno et al. [4] provide recommendations of procedures related to chain of custody in forensic anthropology in Brazil, detailing aspects relevant to keeping the chain of custody in all the steps of the investigation and examinations, considering the new Law that was passed in 2019. They also highlight the importance of preserving evidence integrity from its recognition and collection at the scene until its disposal.

The third contribution from this city, Filho [5] brings us two examples of cases where identity was not achieved, discussing the reasons precluding it. The inexistence of a centralized missing person database should be stressed here since it is a major shortcoming in the forensic reality in Brazil.

Lemos et al. [6], from Belo Horizonte, report a microwave oven case. It is an illegal destruction of a body using a tire stack where the victim is placed. Then they set the structure on fire using flammable substances. Despite the destruction of the body, identification was achieved through the morphology of sella turcica and aspect of the axis, once confronted with antemortem records.

Another case study is from the team of Marcos Paulo Machado from Rio de Janeiro [7], where the fire is a very popular way of trying to vanish with the remains and evidence and hinder the identification of a body. With the help of statistical analysis based on individualizing characters of a carbonized body, the team was able to establish a positive identification.

From Paraíba, we have a case report by Evelyne Soriano Pessoa and her team [8]. It deals with a case of dismemberment in Northeast Brazil, showing how forensic anthropology can shed light on cases involving the identification of dismembered instances, despite the lack of equipment or involvement of this discipline in all the needed steps of the forensic analysis.

Finally, the third contribution from Belo Horizonte assesses the epidemiological and toxicological profile of officially confirmed suicide victims in a 1 year period [9].

We are delighted about the final format of this issue since it reflects the specificities of forensic anthropology in Brazil, a reality quite different from the one of other countries in Latin America. Yet, the continental dimension of the country, the severity of violence, and the shocking numbers of missing persons are finding a more solid answer from the discipline under question. There are now more forensic anthropology research centres, mainly located at the universities (CEMEL—the Legal Medicine Centre of Ribeirão Preto Medical School at the University of São Paulo, CAAF—the Centre for Forensic Archaeology and Anthropology at the Federal University of São Paulo, CEAF—Centre for Studies in Forensic Anthropology, University of Pernambuco, Recife, among others); identified osteological collections are now systematized and organized, and capacitation/education on the field at the level of postgraduation is now happening and being sought. The Brazilian Association of Forensic Anthropology (ABRAF), which is currently commemorating 10 years, was also a big step forward in developing the discipline. Definitely, Brazil is now on the world map of Forensic Anthropology and its practice.


Eugénia Cunha
National Institute of Legal Medicine and Forensic Sciences, Coimbra, Portugal

eugenia.m.cunha@inmlcf.mj.ptBridget Algee-Hewitt
Centre for Functional Ecology, Department of Life Sciences, University of Coimbra, Coimbra, PortugalCCSRE Center for Comparative Studies in Race and Ethnicity (CCSRE), Stanford University, Stanford, CA, USA

Melina Calmon
Grupo de Pesquisa em Antropologia Forense e Identificação de Pessoas – ANP/CNPq, Lago Sul, Brazil


## References

[1] Araújo RM, Lemos YV, do Nascimento ED, et al. Identification of victims of the collapse of a mine tailing dam in Brumadinho. Forensic Sci Res. 2022;7. 10.1080/20961790.2022.2113623 PMC9930756 36817257

[2] Miamoto P, Uehara C. Personal identification and missing persons initiatives in Santa Catarina state, Brazil: forensic perspectives from 2019 to 2021. Forensic Sci Res. 2022;7. 10.1080/20961790.2022.2060653 PMC9930748 36817239

[3] Silva MC. Forensic data management and database systems in forensic investigations for cases of missing and unidentified persons in Brazil. Forensic Sci Res. 2022;7. 10.1080/20961790.2022.2076994 PMC9930801 36817244

[4] Damascena NP, Silva MC, Deitos A, et al. Recommendations of procedures related to the evidence chain of custody in forensic anthropology in Brazil. Forensic Sci Res. 2022;7. 10.1080/20961790.2022.2076984 PMC9930860 36817245

[5] Filho AT. When identity is not reached: two cases from Brasília. Forensic Sci Res. 2022;7. 10.1080/20961790.2022.2076797 PMC9930747 36817251

[6] Lemos YV, Corradi LM, Silva MC, et al. “Microwave oven” practice in Brazil — case report. Forensic Sci Res. 2022;7. 10.1080/20961790.2022.2067727 PMC9930847 36817249

[7] Machado MP, Graça G, Lima L, et al. Anthropological examination in burned body: a retrospective identification case. Forensic Sci Res. 2022;7. 10.1080/20961790.2022.2125429 PMC9930744 36817230

[8] Araújo MDS, Pereira F, Melo F, et al. Case report of a criminal dismemberment in Northeast Brazil. Forensic Sci Res. 2022;7. 10.1080/20961790.2022.2055828 PMC9930774 36817252

[9] Martins CC, Lemos YV, Teodoro M, et al. Epidemio-toxicological profile of suicidal cases: analysis from a forensic unit in Brazil. Forensic Sci Res. 2022;7. 10.1080/20961790.2022.2113622 PMC9930856 36817255

